# Phenazine-1-carboxamide functionalized mesoporous silica nanoparticles as antimicrobial coatings on silicone urethral catheters

**DOI:** 10.1038/s41598-019-42722-9

**Published:** 2019-04-17

**Authors:** Sirisha Kanugala, Sudhakar Jinka, Nagaprasad Puvvada, Rajkumar Banerjee, C. Ganesh Kumar

**Affiliations:** 1 Department of Organic Synthesis and Process Chemistry, CSIR-Indian Institute of Chemical Technology, Uppal Road, Hyderabad, 500007 India; 2 Department of Applied Biology, CSIR-Indian Institute of Chemical Technology, Uppal Road, Hyderabad, 500007 India; 3 Academy of Scientific and Innovative Research, Ghaziabad, 201002 India

**Keywords:** Nanoparticles, Nanoparticles

## Abstract

Microbial infections due to biofilms on medical implants can be prevented by antimicrobial coatings on biomaterial surfaces. Mesoporous silica nanoparticles (MSNPs) were synthesized via base-catalyzed sol-gel process at room temperature, functionalized with phenazine-1-carboxamide (PCN) and characterized by UV-visible, FT-IR, DLS, XRD spectroscopic techniques, SEM, TEM, TGA and BET analysis. Native MSNPs, PCN and PCN-MSNPs were evaluated for anti-*Candida* minimum inhibitory concentration (MIC), minimum fungicidal concentration (MFC), *Candida albicans* (*C*. *albicans*) biofilms and *C*. *albicans-Staphylococcus aureus* (*S*. *aureus*) polymicrobial biofilm inhibition. PCN-MSNPs were four-fold effective (MIC 3.9 µg mL^−1^; 17.47 µM) and MFC (7.8 µg mL^−1^; 34.94 µM) as compared to pure PCN (MIC 15.6 µg mL^−1^; 69.88 µM) and MFC (31.2 µg mL^−1^; 139.76 µM). PCN-MSNPs inhibited *in vitro C*. *albicans* MTCC 227*-S*. *aureus* MTCC 96 biofilms at very low concentration (10 µg mL^−1^; 44.79 µM) as compared to pure PCN (40 µg mL^−1^; 179.18 µM). Mechanistic studies revealed that PCN induced intracellular ROS accumulation in *C*. *albicans* MTCC 227, *S*. *aureus* MTCC 96 and *S*. *aureus* MLS-16 MTCC 2940, reduction in total ergosterol content, membrane permeability, disruption of ionic homeostasis followed by Na^+^, K^+^ and Ca^2+^ leakage leading to cell death in *C*. *albicans* MTCC 227 as confirmed by confocal laser scanning micrographs. The silicone urethral catheters coated with PCN-MSNPs (500 µg mL^−1^; 2.23 mM) exhibited no formation of *C*. *albicans* MTCC 227 - *S*. *aureus* MTCC 96 and *C*. *albicans* MTCC 227 - *S*. *aureus* MLS -16 MTCC 2940 biofilms. This is the first report on PCN-MSNPs for use as antimicrobial coatings against microbial adhesion and biofilm formation on silicone urethral catheters.

## Introduction

Urethral catheter associated biofilm infections frequently arise from diverse polymicrobial communities including *Candida albicans* (*C*. *albicans*), *Staphylococcus aureus* (*S*. *aureus*), *Pseudomonas aeruginosa*, *Escherichia coli*, etc. Around 70–80% of nosocomial infections are related to the use of urethral catheters^1^. As the catheter is inserted into the bladder through urethra, the chance of developing catheter associated infections enhances 3–7% with each day of catheterization^2^, which accounts to huge financial burden since these nosocomial infections cause serious complications under post-surgical or critical care conditions^3^.

*C*. *albicans* is a dimorphic fungus that exists either as commensal on human mucosal surfaces and as an opportunistic pathogen in immunocompromised patients with an ability to cause biofilm-associated infections ranging from superficial to life threatening implications^4^. However, its hyper-proliferation, biofilm formation and defense escape strategies makes this fungal species drug resistant, in its biofilm condition, accounting to ~30% inpatient deaths^5,6^. *S*. *aureus* is a Gram-positive bacterium that exists as commensal on human surfaces and mucosal tracts and acts as an opportunistic pathogen. It is an important pathogen associated with bacteriuria in patients and studies showed three-fold higher deaths in *S*. *aureus* bacteriuria as compared to without bacteriuria^7^. Various types of infections are associated with multi-species biofilms formation on host surfaces^8^. According to a report, *S*. *aureus* is the most prevalent pathogen in Catheter-Associated Urinary Tract Infections (CAUTI) in children^9^. Catheter associated candidiuria is a significant issue among inpatients^10^. Further, species of *Candida* and *Staphylococcus* form mixed biofilms over various medical devices^11–13^. Therefore, it is mandatory to identify new or improved leads that inhibit clinically significant *C*. *albicans* or mixed biofilms of *C*. *albicans* - *S*. *aureus*. Equally there is a need for safe and biocompatible drug carriers that enable controlled drug release and increased bioavailability thus lowering the therapeutic dosage.

Microbial phenazines are pigmented, redox-active, nitrogenous aromatic compounds with metabolic, ecological and evolutionary significance^14^. All these distinctive features make them attractive microbial metabolites. More than 100 natural and 6000 synthetic phenazines are reported to date exhibiting promising bioactivities including antimicrobial^15–17^, anticancer^18^, anti-parasitic^19^, insecticidal^20^ and biocontrol properties^21,22^. In spite of greater potential and scope for phenazines as antimicrobial agents, no leads have emerged in this regard except one synthetic phenazine analogue of riminophenazine family, 2-chloro-anilino-5-*p*-chlorophenyl-3,5-dihydro-3-isopropyl iminophenazine (B663) which was active against *Mycobacterium leprae* with WHO approval and marketed under the trade name, Lamprene or Clofazimine^23^.

Among the drug-delivery systems, mesoporous silica nanoparticles (MSNPs) have gained attention as smart nanocarriers with their unique tunable features like pore size, volume, surface area, controlled, stimuli-dependant drug delivery, stability to organic solvents, biocompatibility and FDA approval^24–26^. Moreover, limitations of conventional therapeutics like poor stability, solubility, non-specificity, low pharmacokinetic profiles, and narrow pharmaceutical window were resolved by MSNPs^27^. MSNPs functionalized to small molecules exhibited enhanced antimicrobial properties^28^.

However, there are no reports showing the effects of MSNPs functionalized with microbial derived products on pathogenic biofilms. In one of the study from our laboratory, Kocuran, a microbial exopolysaccharide derived from *Kocuria rosea* strain BS-1, functionalized on silver glyconanoparticles showed potential antimicrobial and anti-adhesive properties, were demonstrated as antimicrobial coatings on silicone urethral catheters that prevented bacterial adhesion and biofilm formation^29^. However, according to our knowledge, there are no reports on effects of microbial-derived small molecule functionalized silica nanoparticles against mixed biofilms. In this context, the current report is the first study on the phenazine functionalization or conjugation to nanoparticles as an antimicrobial coating on urethral catheters against *C*. *albicans* unispecies biofilms or in mixed biofilms with *S*. *aureus*.

## Experimental Details

### Materials

All chemicals of analytical grade were procured from Sigma-Aldrich (St. Louis, MO, USA) unless otherwise stated and were used as such without any further purification. Absolute ethanol was procured from Merck KGaA, Darmstadt, Germany. All culture media were purchased from HiMedia Laboratories Pvt., Mumbai, India.

### Synthesis of mesoporous SiO_2_ nanoparticles

MSNPs were synthesized by mixing cetyltrimethylammonium bromide (CTAB) with ethanol and water (1:1 ratio)^30^. Ammonia was added to this solution and stirred vigorously for 20 min to catalyze tetraethoxysilane (TEOS) polymerization in the next step. TEOS (0.1%) was added to the ammonic slurry as a precursor and stirred well for 3 h. The precipitate formed was washed with dilute HCl by overnight stirring. The entire mixture was centrifuged at 12000 × *g* for 20 min followed by water washing the precipitate for six times to remove residual CTAB and other reagents, followed by overnight drying at 50 °C. The resulting MSNPs were further characterized employing various analytical techniques.

### Extraction and purification of PCN from *Pseudomonas aeruginosa* CGK-KS-1

*Pseudomonas aeruginosa* strain CGK KS-1 (GenBank Accession No. KY203649) was grown in Luria-Bertani broth at 30 °C, 150 rpm for 48 h. The cell-free supernatant was collected by centrifugation at 8,500 × g at room temperature followed by ethyl acetate extraction and crude extract was collected in the ethyl acetate layer, which was subjected to rotary vacuum evaporation (Rotavapor R-205; Büchi Labortechnik AG, Flawil, Switzerland) to obtain a dried crude extract. This crude extract was subjected to preparative TLC using TLC silica gel 60 F_254_ plate (Merck, Germany) for metabolic profiling using mobile phase of methanol:chloroform (10:90, v/v). The major metabolite (PCN) on the TLC plate was recovered under UV Transilluminator Cabinet 4 (Camag, Wilmington, NC, USA), dissolved in methanol and filtered through 0.22 µm nylon syringe filters (Millex-HN, Millipore Corporation, Billerica, MA, USA) to remove silica particles and collect the pure PCN metabolite^31^.

### Field Emission Scanning Electron Microscopy (FE-SEM) and Transmission electron microscopy (TEM) studies

The synthesized MSNPs were examined by Field Emission Scanning Electron Microscope (FE-SEM) and Transmission Electron Microscope (TEM) for determining their morphology, average size, loading of PCN into the pores of MSNPs and surface coating of PCN onto MSNPs. The FE-SEM images of amorphous MSNPs were captured on JSM 7610 F Schottky Field Emission Scanning Electron Microscope (JEOL USA Inc., MA, USA) operated at accelerating voltage of 100 kV. TEM analysis was performed on MSNPs dispersed in methanol followed by sonication in a water bath, placing over copper grids and observed under JEM-2100 Transmission Electron Microscope (JEOL Ltd., Tokyo, Japan) operated at accelerating voltage of 200 kV.

### Fourier transform-infrared spectroscopy (FT-IR) studies

FT-IR spectra of both the MSNPs and PCN-MSNPs were recorded on Thermo Nicolet Nexus 670 FT-IR spectrophotometer (Thermofisher Scientific Inc., Madison, WI, USA) in the wavenumber region of 400–4000 cm^−1^ at a resolution of 4 cm^−1^. The binding of PCN to MSNPs was monitored by shift in the wavenumber peaks as compared to that of pure PCN, based on our previous report^31^.

### BET analysis

MSNPs and PCN-MSNPs were subjected to BET analysis for determining specific surface area, pore size and pore volume using BELSorb mini II (MicrotracBEL Corporation, Osaka, Japan) by degassing at 200 °C under nitrogen flow for about 2 h, prior to measurement. The nitrogen adsorption/desorption (ADS/DES) data were recorded at the liquid nitrogen temperature (77 K). The specific surface area was calculated using the Brunauer–Emmett–Teller (BET) equation. The micropore radius distributions were determined by micropore analysis method. The average pore size was derived from the peak maxima of the pore size distributions. The mesopore diameter distribution was determined using Barrett–Joyner–Halenda method from the nitrogen adsorption values. The volume of liquid nitrogen adsorbed at *P/P*_0_ = 0.99 was reported as pore volume.

### Dynamic light scattering (DLS) studies

DLS studies were carried out to determine the hydrodynamic diameter of MSNPs and PCN-MSNPs (1 mg mL^−1^) in sterile PBS at pH 7.4. These suspensions were analyzed for their size and zeta potential measurements using Litesizer^TM^ 500 (AntonPaar GmbH, Graz, Austria). The refractive index and viscosity of ultrapure water were referred for size measurements of MSNPs and PCN-MSNPs. The intensity distributions gave the hydrodynamic diameters of MSNPs and PCN-MSNPs. To measure the long-term stability of the PCN coating on MSNPs, the DLS study was performed periodically every month for six months.

### Thermogravimetric analysis (TGA)

Thermogravimetric analysis (TGA) of synthesized MSNPs and PCN-MSNPs was performed using a thermal analyzer (TGA Q-500, TA Instruments, MN, USA) interfaced with TRIOS software. The synthesized MSNPs and PCN-MSNPs (20 mg) were heated from room temperature to 800 °C at a heating rate of 10 °C min^−1^. The instrument was purged with air at a flow rate of 10 mL min^−1^. Later it was calibrated for temperature and heat flow using α-alumina before measurements.

### X-ray diffraction (XRD) analysis

The phase purity and crystalline structure of the synthesized MSNPs were analyzed by X’Pert3 powder X-ray diffractometer (Malvern PANalytical BV, Almelo, The Netherlands) using Cu-Kα radiation of l = 1.5418 Å from 2 y = 2–10° low angle with a step width of 0.1 The characteristic crystalline lattice parameters of MSNPs were derived using the following equation: a_o_ = 2d_100_3^1/2^.

### Functionalization of MSNPs with PCN

The synthesized MSNPs (15 mg) were sonicated in Milli Q water to form aqueous suspension of MSNPs, which was mixed vigorously with the freshly purified PCN (5 mg mL^−1^). This mixture was stirred for 6 h to facilitate the loading and binding of PCN to MSNPs at room temperature. The unbound PCN was removed by centrifugation at 8000 × *g* followed by discarding the supernatant. The binding of PCN to MSNPs was confirmed by shift in the two characteristic peaks in the UV spectrum of phenazines^32,33^.

### *In vitro* release of PCN

The *in vitro* release of PCN from the PCN-MSNPs was analyzed at two different pH conditions (pH 5.2 and pH 7.4), which was monitored by scanning in the wavelength range from 190 to 700 nm on a UV-visible double beam spectrophotometer (Lambda 25, PerkinElmer, Shelton, CT, USA), at an interval of 4 h for a total period of 40 h. To this, 3 mg mL^−1^ of PCN-MSNPs was suspended in PBS buffer and maintained at pH 5.2 and pH 7.4 separately at 37 °C under shaking conditions. The recorded absorbance values were compared with PCN standard curve to derive the percentage of PCN released from PCN-MSNPs at every time interval. Finally a plot was constructed showing percentage of the PCN release at both pH 5.2 and pH 7.4 against various time intervals. All these release studies were performed in triplicates and standard deviation is represented for each data point.

### PCN standard curve

The purified PCN was dissolved in PBS to attain different concentrations ranging from 0.4–2 mg mL^−1^ and scanned in the wavelength range from 190 to 700 nm on a Lambda 25 UV-visible double beam spectrophotometer (PerkinElmer). A standard curve of PCN was constructed by plotting various concentrations of PCN against corresponding absorbance values. All the absorbance readings were measured in triplicates and the average values were used to plot the standard curve.

### Antifungal activity assay

The antifungal activity of the purified PCN and PCN-MSNPs was performed by agar well diffusion method^34^. All the *Candida* strains used in the study, namely *C*. *albicans* MTCC 227, *C*. *albicans* MTCC 1637, *C*. *albicans* MTCC 3017, *C*. *albicans* MTCC 3018, *C*. *albicans* MTCC 4748, *C*. *albicans* MTCC 7315, *C*. *glabrata* MTCC 3019, *C*. *parapsilosis* MTCC 1744, *C*. *krusei* MTCC 3020 and *Issatchenkia hanoiensis* MTCC 4755 were procured from Microbial Type Culture Collection and Gene Bank (MTCC), CSIR-Institute of Microbial Technology, Chandigarh, India. These *Candida* strains were cultured overnight in Muller-Hinton broth (MHB) at 37 °C for 24 h. Muller-Hinton agar (MHA) plates were seeded with 1 × 10^6^ cells (equivalent to 0.5 McFarland standard) for each *Candida* strain and wells were made with cork borer. The wells were loaded with various concentrations of PCN and PCN-MSNPs (250 to 0 µg mL^−1^; 1.11 to 0 mM). Minimum inhibitory concentration (MIC) of PCN or PCN-MSNPs represents the well containing the least concentration exhibiting the zone of inhibition. The native MSNPs (250 to 0 µg mL^−1^) and Miconazole (MZ) standard (250 to 0 µg mL^−1^; 600.78 to 0 µM) served as negative and positive controls, respectively. This antifungal assay was performed in triplicates and their mean were used for determining the MIC values.

### Minimum fungicidal concentration (MFC) assay

The viability of various *Candida* strains upon treatment with PCN and PCN-MSNPs was studied and their minimum fungicidal concentration (MFC) was determined based on the fungicidal assay with slight modifications^35^. The MFC assay was performed on those *Candida* strains that were susceptible in the antifungal assay. MFC determination was carried out in sterile 2.0 mL microfuge tubes with MHB. PCN and PCN-MSNPs were added at a dose range of 62.5 to 0.9 µg mL^−1^ (279.98 to 4.03 µM) to each *Candida* strain and incubated at 30 °C for 24 h. MFC is the lowest concentration of PCN or PCN-MSNPs required to kill a particular *Candida* strain. The native MSNPs (250 to 0 µg mL^−1^) and MZ standard (250 to 0 µg mL^−1^; 600.78 to 0 µM) served as negative and positive controls, respectively. All the experiments were repeated thrice and their mean values were represented.

### Polymicrobial inhibition assay

The effect of PCN and PCN-MSNPs against a mixture of *C*. *albicans* MTCC 227 and the bacterial strains, *S*. *aureus* MTCC 96 or *S*. *aureus* MLS-16 MTCC 2940 was assessed by modified agar well diffusion method^34^. These pathogenic yeast and bacterial strains were cultured in MHB separately and diluted to optical density (OD) of 0.1. Equal volumes of *C*. *albicans* MTCC 227 + *S*. *aureus* MTCC 96 and *C*. *albicans* MTCC 227 + *S*. *aureus* MLS-16 MTCC 2940 were mixed and spread over MHA plates. Different concentrations of PCN and PCN-MSNPs were loaded into the wells at concentrations of 250 to 0 µg mL^−1^ (1.11 to 0 mM) and incubated at 37 °C for 24 h. The native MSNPs (250 to 0 µg mL^−1^) and MZ standard (250 to 0 µg mL^−1^; 600.78 to 0 µM) served as controls. The experiments were carried out in triplicates and the diameters of zone of inhibition versus concentration of PCN and PCN-MSNPs against various *Candida* strains were plotted as an antibiogram.

### *Candida* biofilm inhibition assay

The PCN and PCN-MSNPs were evaluated for their *Candida* biofilm inhibition property in microtiter plates^36^ against various *Candida* strains. The entire panel of test *Candida* strains were incubated in 96 well plates containing MHB at 37 °C for 48 h to allow biofilm formation. Later the biofilms were treated with PCN and PCN-MSNPs at a dose range of 0, 2, 4, 8, 16 and 20 µg mL^−1^ (0, 8.95, 17.91, 35.83, 71.67, 89.59 µM) for 24 h. The unattached cells were removed by washing with PBS (pH 7.4), followed by staining of biofilms with 40 µL of 0.1% crystal violet solution for 45 min. The stained plates were washed with sterile distilled water and were dried overnight at room temperature to remove the excess water, if any. Then the stained biofilms were eluted and solubilized in 120 µL of 95% ethanol, whose absorbance was measured at 540 nm using Infinite M200Pro microtitre plate reader (Tecan Group Ltd., Männedorf, Switzerland). Based on the absorbance values of treated and control samples, the half maximal inhibitory concentration (IC_50_) values of PCN and PCN-MSNPs were calculated in µM. The native MSNPs (0, 2, 4, 8, 16 and 20 µg mL^−1^) and MZ standard (0, 2, 4, 8, 16 and 20 µg mL^−1^; 0, 4.80, 9.61, 19.22, 38.45 and 48.06 µM) served as negative and positive controls, respectively. All the experiments were performed in triplicates and the mean values were represented with standard deviations.

### Polymicrobial biofilm inhibition assay

The effect of PCN and PCN-MSNPs on mixed biofilms was studied by a previously described method^12^ with minor modifications. The overnight cultures of *C*. *albicans* MTCC 227, *S*. *aureus* MTCC 96, *S*. *aureus* MLS-16 MTCC 2940 were diluted with MHB medium to OD of 0.1 at 600 nm, mixed and loaded into each well of 96 well microtitre plate and incubated at 37 °C for 24 h. The medium was discarded and the formed biofilms were stained with 0.1% crystal violet solution for 45 min. Solubilization and elution of the stained biofilms was performed with 95% ethanol and then the plates were air-dried to remove excess water, if any. The absorbance values of the solubilized biofilms were recorded at 540 nm on an Infinite M200Pro microtitre plate reader (Tecan). All the mixed biofilm studies were performed in triplicates and average values were considered for determining the standard deviations.

### Ergosterol quantification assay

A panel of *Candida* strains susceptible in antifungal activity, were used for observing the effect of PCN on their total sterol content. The sterols from the test *Candida* strains were isolated by using total sterol extraction method^37^ with minor modifications. Initially, MHB (50 mL) spiked with various concentrations of PCN (0, 4, 8, 16 and 32 µg mL^−1^; 0, 17.91, 35.83, 71.67, 143.3 µM) was inoculated with single colonies of test *Candida* strains. Untreated cells (0 µg mL^−1^) were used a control. The treated culture medium was incubated at 37 °C for 20 h with agitation at 150 rpm. The cells were collected at stationary phase by centrifugation at 8200 × *g* for 6 min followed by washing the pellet twice with sterile distilled water. The net wet weight of each *Candida* pellet was weighed and recorded. To each *Candida* pellet, 5 mL of 25% alcoholic potassium hydroxide solution was added and mixed. Each of these *Candida* suspensions were collected in sterile glass vials and incubated at 85 °C for 60 min in a water bath. After cooling to room temperature, mixture of sterile distilled water and n-heptane (1:3) was added to each *Candida* suspension and vortexed vigorously for 5 min for the extraction of intracellular sterols into the n-heptane layer. From each *Candida* strain bilayer, the heptane layer with sterols was collected carefully and stored at −20 °C for 24 h. The sterol suspension (30 µL) of each *Candida* strain was diluted with 100% ethanol in 1:5 ratio and scanned from 230–310 nm wavelength using UV-visible spectrophotometer (PerkinElmer) to observe the four characteristic peaks corresponding to ergosterol and dehydroergosterol. The following equations were used to calculate the ergosterol content of each test *Candida* strain as percentage of wet weight of the cell pellet.

$$ \% \,{\rm{ergosterol}}+ \% 24(28)\,{\rm{DHE}}=[({\rm{A}}281.5/290)\times F]/{\rm{pellet}}\,{\rm{weight}}$$

$$ \% \,24(28)\,{\rm{DHE}}=[({\rm{A}}230/518)\times F]/{\rm{pellet}}\,{\rm{weight}}$$

$${\rm{F}}={\rm{Dilution}}\,{\rm{factor}}\,{\rm{for}}\,{\rm{dissolving}}\,{\rm{in}}\,{\rm{ethanol}}$$

$$290={E}\,{\rm{value}}\,({\rm{percent}}\,{\rm{per}}\,{\rm{centimeter}})\,{\rm{for}}\,{\rm{crystalline}}\,{\rm{ergosterol}}$$

$$518=E\,{\rm{value}}\,({\rm{percent}}\,{\rm{per}}\,{\rm{centimeter}})\,{\rm{for}}\,{\rm{crystalline}}\,{\rm{dehydroergosterol}}$$

All the experiments were performed in triplicates and mean values were considered for calculating the standard deviations. The statistically significant differences in ergosterol content in various treatment groups at 95% confidence level were determined using ANOVA in GraphPad PRISM software ver. 8.0.2 (GraphPad Software, Inc, La Jolla, CA, USA).

### ROS quantification assay in *C*. *albicans* MTCC 227

Several antifungals cause ROS induced cell death in fungi. Considering this fact, the intracellular ROS accumulation was measured in *C*. *albicans* MTCC 227 by 2′,7′-dichlorofluorescein diacetate (DCFH-DA)-dependant fluorometric assay^38^. Initially, the *C*. *albicans* MTCC 227 biofilm development in 96-well microtiter plate was achieved by culturing the strain at 37 °C for 24 h. Later, these biofilms were incubated for 24 h with PCN, PCN-MSNPs and MZ at a concentration of their corresponding MFCs (15.6, 7.8 and 7.8 µg mL^−1^; 69.88, 34.94 and 18.74 µM), respectively. As the catheters were coated with 500 µg mL^−1^ of PCN-MSNPs, the effect of MSNPs alone on the ROS accumulation was measured, to know if there was any effect of MSNPs at a dose of 500 µg mL^−1^, where untreated cells served as control. All the groups were treated with 10 µM of DCFH-DA and incubated in dark for 24 h to allow the reaction of PCN induced cells with DCFH-DA for determining the intracellular ROS accumulation. The resultant fluorescent product was measured at excitation and emission wavelengths of 485 nm and 535 nm on Infinite M200Pro microtiter plate reader (Tecan). The resulting fluorescence intensities were plotted against concentration of PCN and MZ standard. All the measurements were performed in triplicates and the mean values were considered for the calculation of standard deviation. Statistically significant differences in data variances (p < 0.05) in the amount of ROS accumulated in different treatment groups were determined, using one-way ANOVA, in GraphPad PRISM software ver. 8.0.2 (GraphPad Software, Inc, La Jolla, CA, USA). Groups with significant differences were analyzed by Turkey’s multiple comparisons test.

### ROS quantification assay in *S*. *aureus* MTCC 96 and *S*. *aureus* MLS-16 MTCC 2940 biofilms

The effect of PCN, MSNPs and PCN-MSNPs on intracellular ROS accumulation in *Staphylococcus* strains was studied by nitroblue tetrazolium (NBT) assay^39^. *S*. *aureus* MTCC 96 and *S*. *aureus* MLS-16 MTCC 2940 were cultured in MHB, diluted to achieve 0.5 McFarland standard and dispensed into the wells of 96 well microtitre plate. After treatment with PCN, MSNPs and PCN-MSNPs (MIC value 3.9 µg mL^−1^; 17.47 µM) for 3 h, all the strains were stained with nitroblue tetrazolium chloride (0.1% in PBS) at 37 °C for 40 min. As the catheters were coated with 500 µg mL^−1^ of PCN-MSNPs, the effect of MSNPs alone on the ROS accumulation was measured, to know if there is any effect of MSNPs at a dose of 500 µg mL^−1^, where untreated cells served as control. Initially, the NBT was yellowish in color and on reaction with PCN induced ROS formed a blue formazan product that was spectrophometrically read at 570 nm. All the experiments were performed in triplicates and the mean values were considered for the calculation of standard deviation. The absorbance which corresponded to the percentage of intracellular ROS accumulated versus concentration of PCN was plotted. Statistically significant differences in data variances (p < 0.05) in the ROS accumulation due to different treatment groups were determined, using one-way ANOVA, in GraphPad PRISM software ver. 8.0.2 (GraphPad Software, Inc, La Jolla, CA, USA). Groups with significant differences were analyzed by Turkey’s multiple comparisons test.

### Confocal laser scanning microscopy

The effect of PCN-MSNPs on cell membrane integrity and viability of *C*. *albicans* MTCC 227 cells was assessed by staining with LIVE/DEAD^®^ *Bac*Light^™^ kit (Molecular Probes, Eugene, OR, USA) and further visualized by confocal laser scanning microscopy (CLSM). Initially, *C*. *albicans* MTCC 227 cells (OD_600_ = 0.01) were cultured in MHB in two CLSM dishes (SPL Scientific, Gyeonggi-do, South Korea) at 37 °C for 18 h. Later, one group of *C*. *albicans* MTCC 227 was treated with PCN (15.6 µg mL^−1^; 69.88 µM) for 24 h and another group was untreated. To these groups, 1 µL each of SYTO 9 and propidium iodide were added in dark, mixed and incubated for 40 min at room temperature. The stained *C*. *albicans* MTCC 227 cells were fixed with 0.1% formaldehyde for 20 min. The fixed cells were then visualized by Eclipse Ti confocal laser scanning microscope (Nikon Corporation, Tokyo, Japan) interfaced with an argon ion laser at 480–490 nm wavelength for excitation and 500–635 nm band pass filter wavelength for emission. CLSM images were processed by Ti Control software ver. 4.4.4 and the scale bar for each image was 10 μm.

### Elemental analysis using Inductively Coupled Plasma-Optical Emission Spectrometry (ICP-OES)

*C*. *albicans* MTCC 227 was cultured in two groups in MHB. One group was untreated and used as control, while the another group was treated with 15.6 µg mL^−1^ (69.88 µM) of purified PCN for 24 h. Later, both the groups were centrifuged at 8000 × *g* and the supernatants were filtered through 0.2 µm syringe filters. The amount of Na^+^, K^+^ and Ca^2+^ ions released into both the supernatants due to cell lysis was quantified using iCAP^TM^ 6500 ICP-OES DUO Analyzer (Thermo Fisher Scientific, Cambridge, UK). Standards of Na^+^, K^+^ and Ca^2+^ ions (1 and 5 ppm) were prepared by serial dilution in sterile Milli Q water. The quantification of Na^+^, K^+^ and Ca^2+^ ions were carried out at 588.9, 766.4 and 317.9 nm, respectively. This analysis was performed thrice and the concentration of various ions were calculated using linear regression method.

### Evaluation of anti-biofilm activity of PCN-MSNPs coating on silicone urethral catheters

Silicone urethral catheters were coated with PCN-MSNPs using simple dispersion method^29^. A silicone urethral catheter (RÜSCH Sterile, Teleflex Medical Sdn, Perak, Malaysia) was cut into 4 cm long pieces, which were incubated in PCN-MSNPs solution (500 µg ml^−1^; 2.23 mM) at 37 °C for 24 h for effective coating. The coated catheter pieces were dried well to form the antimicrobial coating. The performance of this antimicrobial coating was assessed in a time-dependant manner at periodic interval of 24 h for up to 72 h against *C*. *albicans* MTCC 227*-S*. *aureus* MTCC 96 and *C*. *albicans* MTCC 227*-S*. *aureus* MLS-16 MTCC 2940 mixed biofilms. Coated and uncoated catheter pieces were incubated for biofilm formation in overnight grown mixed cultures of *C*. *albicans* MTCC 227*-S*. *aureus* MTCC 96 and *C*. *albicans* MTCC 227*-S*. *aureus* MLS-16 MTCC 2940 for 24, 48 and 72 h, at 37 °C with agitation at 150 rpm. After corresponding incubation period, each catheter piece were washed with distilled water twice and stained with crystal violet (0.1%) solution for 15 min. The dye from each PCN-MSNP coated catheter piece was extracted with the solubilization reagent (0.04 N HCl - isopropanol and 0.25% SDS). The collected dye at different time intervals was loaded in wells of 96 well microtitre plate and read at 590 nm employing microtitre plate reader (Tecan), where the absorbance was directly proportional to biofilm formation. The anti-biofilm effect of PCN-MSNPs coating was expressed as percentage of biofilm formation as a function of time. All the coating studies were performed in triplicates and mean values are represented. Statistically significant differences in data variances (p < 0.05) of biofilm growth percentage at different time points were determined using one-way ANOVA, in GraphPad PRISM software ver. 8.0.2 (GraphPad Software, Inc, La Jolla, CA, USA). Groups with significant differences were analyzed by Turkey’s multiple comparisons test.

### *In vitro* cytotoxicity assay

The NIH-3T3 cell line derived from mouse embryonic fibroblasts (ATCC No. CRL-1658) were employed for 3-(4,5-dimethylthiazol-2-yl)-2,5-diphenyltetrazolium bromide (MTT) based cytotoxicity assay^40^. The cells were cultured in DMEM supplemented with 10% FBS, 2 mM L-glutamine, penicillin and streptomycin (0.05%) and maintained in a humidified incubator with 5% CO_2_ at 37 °C. The cells were seeded in microtiter plates and were allowed to form a monolayer. PCN-MSNPs concentrations (0.1 to 100 µM) were added to the cells in triplicates and incubated for 24 h. MTT was added to the treated cells for 3 h, followed by addition of 100 µL of DMSO. Along with the PCN-MSNPs treatment, Mitomycin-C and untreated cells were run in parallel as positive and negative controls, respectively. The absorbance was recorded at 570 nm. Average values of triplicates were considered to plot a graph with number of cells versus absorbance.

### Statistical analysis

The results were presented as mean values ± standard error of mean. The experimental data were analyzed by one-way ANOVA using GraphPad PRISM version 6.0 (GraphPad Software, Inc, La Jolla, CA, USA). The values of P < 0.05, were accepted as level of significance.

## Results and Discussion

The mesoporosity of the synthesized MSNPs was confirmed by BET surface area analysis with the following results: Specific surface area = 83.7 m^2^ g^−1^; pore volume = 0.13 cm^3^ g^−1^ and pore diameter = 6.43 nm. The binding of PCN to the MSNPs was confirmed by BET surface area analysis with the following results: Specific surface area = 29.7 m^2^ g^−1^; pore volume = 0.043 cm^3^ g^−1^ and pore diameter = 5.86 nm^41^.

### Characterization studies

**Figure 1:**
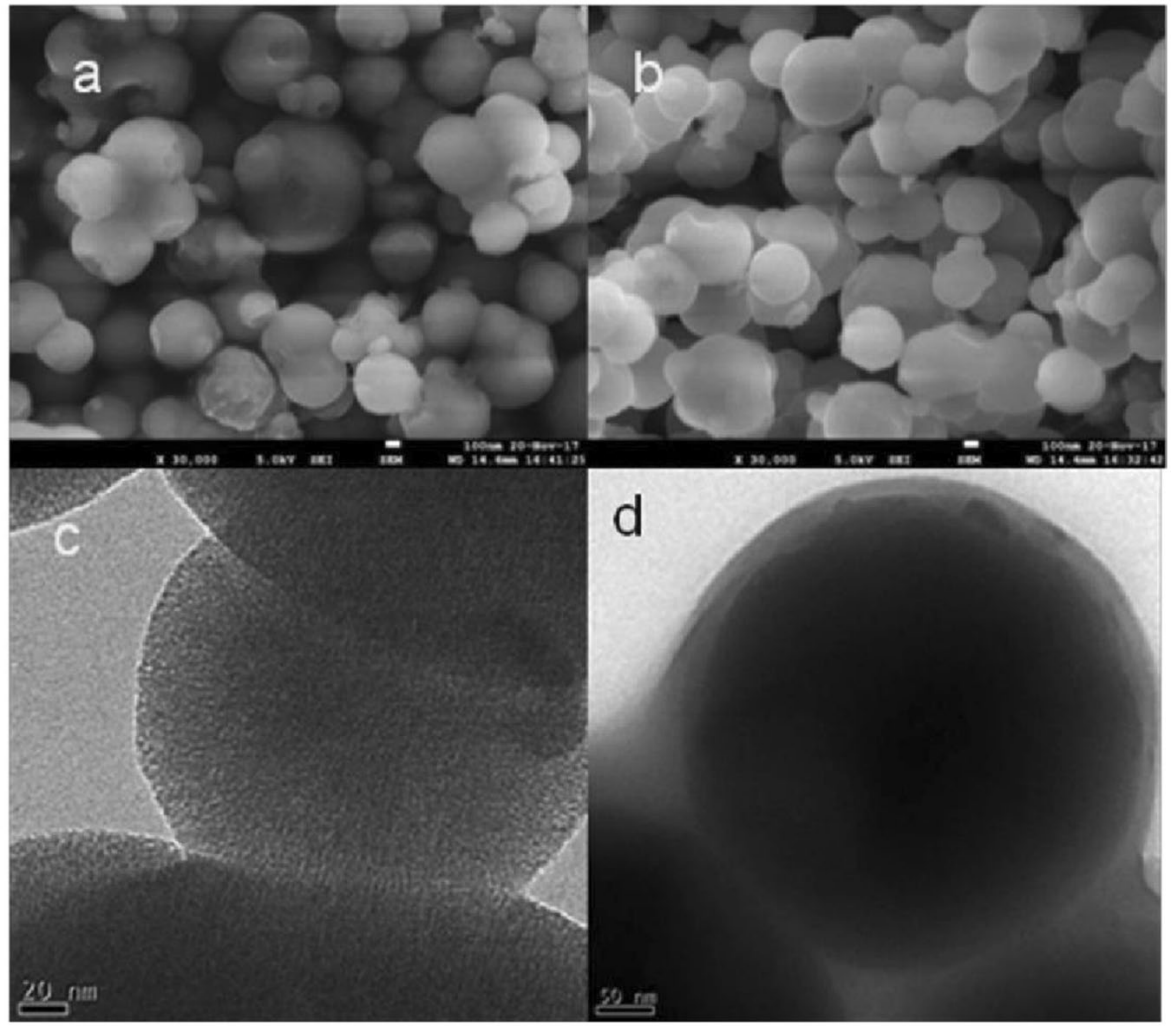
Scanning and Transmission electron micrographs of synthesized mesoporous silica nanoparticles. (**a**) Scanning electron micrographs of mesoporous silica nanoparticles without PCN loading. (**b**) Scanning electron micrographs of mesoporous silica nanoparticles showing pores filled with PCN. (**c**) Transmission electron micrographs of mesoporous silica nanoparticles (average size of 193 nm). (**d**) Transmission electron micrographs of PCN functionalized mesoporous silica nanoparticles (average size of 234 nm).

To determine the size and shape, the MSNPs were examined by FE-SEM and TEM and the corresponding micrographs (Fig. 1) showed that the formed MSNPs and PCN-MSNPs were uniform, monodispersed and spherical in shape. The TEM micrographs revealed that the pores of MSNPs were also filled with PCN. The low angle powder XRD data showed characteristic Bragg diffraction peak for MSNPs with a 2θ value of 2.1° corresponding to (100) facet peak of hexagonally ordered pore channeling (Supplementary Fig. S1).

**Figure 2:**
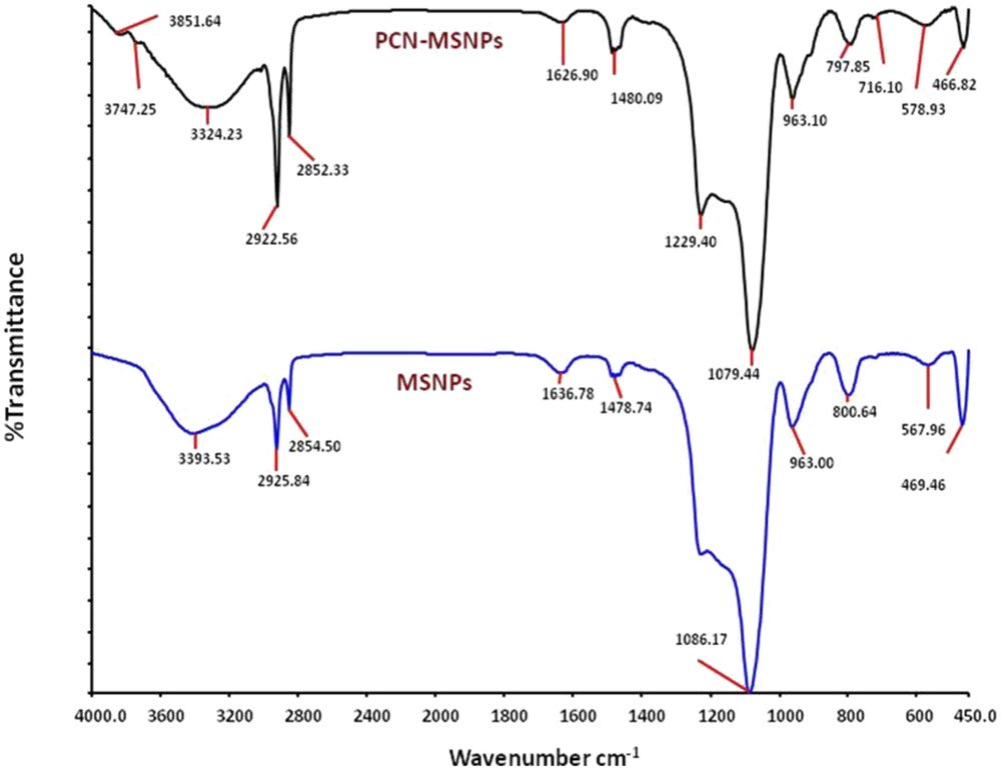
Fourier transform infrared (FT-IR) spectra. (**A**) Phenazine-1-carboxamide functionalized mesoporous silica nanoparticles showing characteristic peaks of aromatic C=C stretching and C=O stretching of PCN. (**B**) Synthesized mesoporous silica nanoparticles showing characteristic Si-O-Si and Si-OH stretchings of SiO_2_ nanoparticles.

The MSNPs were functionalized with freshly purified PCN to record the FT-IR spectrum. FT-IR spectrum of synthesized MSNPs (Fig. 2) showed characteristic signal transmittance of silica nanoparticles^40^. The wavenumbers of 469.46, 800.64, 1086.17 cm^−1^ were attributed to Si-O-Si bending, Si-O-Si symmetric stretching and Si-O-Si asymmetric stretching vibrations, respectively. The bending and stretching vibrations of Si-OH bonds corresponded to transmittance at 963.00 cm^−1^. The characteristic peaks at 3393.53 cm^−1^ indicated the presence of surface hydroxyls of Si-OH in the synthesized MSNPs. The H-O-H bond vibration of water molecule was indicated by 1636.78 cm^−1^. In the PCN-MSNPs spectrum (Fig. 2), the peak at 1636.78 cm^−1^ disappeared suggesting the reduction in H-O-H bond by PCN. The peaks at 469.46, 800.64 and 1086.17 cm^−1^ were either shifted or their intensities were lowered. The shift of signal from 3393.53 cm^−1^ to 3324.23 cm^−1^ and its increased transmittance showed the reduction of surface hydroxyls and their modification by NH from PCN as indicated by NH stretching at 3747.25 cm^−1^.

In our previous study^31^, the FT-IR spectrum of the pure PCN showed characteristic peaks in the range of 2900–3000, especially at wavenumber of 2922 cm^−1^, which is closer to the peaks resulting from CTAB improper washing. The wavenumber peak at 2922 cm^−1^ attributed to aromatic CH stretching. While in the FT-IR spectrum (Fig. 2), PCN-MSNPs showed characteristic peaks of PCN in the wavenumber range of 2900–3000 cm^−1^ along with that of native MSNPs. However, in the FT-IR spectra of native MSNPs, the peaks corresponding to CTAB residues were also observed as reported earlier^42^. We presume that PCN was successfully functionalized as evident from the characteristic peak observed at a wavenumber of 2922 cm^−1^ in the revised spectrum of PCN-MSNP. This aspect was further confirmed through thermogravimetric analysis (TGA) of MSNPs and PCN-MSNPs.

**Figure 3:**
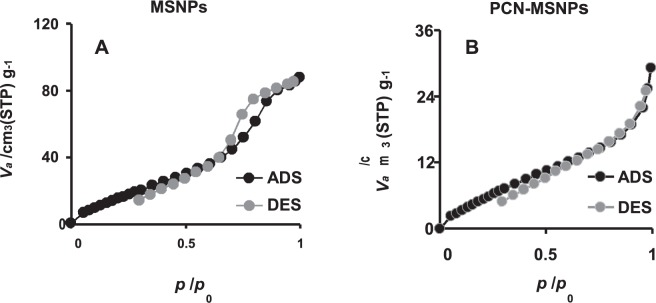
Nitrogen sorption isotherms of synthesized mesoporous silica nanoparticles ADS - Adsoprtion of N_2_, DES - Desorption of N_2_. (**A**) Characteristic type IV hysteresis curves showing mesoporosity of synthesized silica nanoparticles. (**B**) Shift in type IV characteristic curve showing PCN functionalization on silica nanoparticles.

The average size of synthesized MSNPs and PCN-MSNPS based on DLS was 193 nm and 234 nm, suggesting the shape of nanoparticles to be uniform and monodispersed (Supplementary Fig. S2). The zeta potential values of MSNPs and PCN-MSNPs were 2.5 mV and 14 mV, respectively. The nitrogen adsorption-desorption isotherms of MSNPs and PCN-MSNPs shown in Fig. 3A depicts the characteristic type IV hysteresis loops of mesoporous silica nanoparticles^41^. According to BET analysis (Fig. 3), the volumes of MSNPs and PCN-MSNPs were 0.13 and 0.043 cm^3^ g^−1^, respectively. Based on the comparative analysis of the pore volumes, it could be deduced that an amount of PCN corresponding to a volume of 0.087 cm^3^ g^−1^ of each nanoparticle was loaded with PCN with a drug loading capacity of 53%. The functionalization of PCN to MSNPs resulted in reduced pore size indicating the successful loading of PCN into the mesopores. The TGA of MSNPs showed the characteristic curve corresponding to the temperature vs. weight percentage plots, with a slight decrease in the weight percentage owing to the removal of CTAB at temperature above 250 °C (Supplementary Fig. S3). The curve representing the PCN-MSNPs showed a considerable decrease in weight percentage that could possibly be attributed to the melting of both PCN and residual CTAB. The melting of PCN started after 220 °C and the curve observed confirmed the efficient loading of PCN into the synthesized MSNPs, as evident from the characteristic melting and degradation patterns of PCN (Supplementary Fig. S3).

UV-visible spectrum of the purified PCN showed characteristic absorbance at 294 and 431 nm (Supplementary Fig. S4). After functionalization of PCN to MSNPs, a shift in both the characteristic absorbance peaks of PCN was observed. Only PCN was being released into the medium which was observed by UV-visible spectrum, while there was no characteristic absorbance peaks observed for MSNPs. DLS studies showed that MSNPs were stable up to 4 months without any disintegration to form smaller and soluble particles, which is in correlation with our previous study^43^. The successful coating of phenazine-1-carboxamide on mesoporous silica nanoparticles (PCN-MSNPs) was confirmed by the change in the color of catheter surface.

### *In vitro* release of PCN

The *in vitro* PCN release profile from PCN-MSNPs was monitored for 40 h at regular intervals of 4 h each (Supplementary Fig. S5). PCN release (85%) was higher at pH 5.2 as compared to pH 7.4 where the release was only 46%, which was in a slow, stable and controlled pattern, thus increasing the bioavailability and lowering the effective dosage by two to three folds, which was in a similar trend to that of our previous study that showed slow and controlled drug release from MSNPs in acidic conditions^43^. Only PCN was being released into the medium which was observed by UV absorbance spectra. Especially, a slow release pattern of PCN at pH 7.4 was observed suggesting that it remained bound to MSNPs under physiological conditions thus exhibiting promising lower effects on the normal cells and tissues. At pH 5.2, which was acidic in nature mimicked the infected tissues, the release profile of PCN was very stable and controlled. This could plausibly be due to a simple change in redox potential of amino groups of PCN, which enabled the release of PCN. Overall, the *in vitro* release profile indicated that functionalization of PCN to MSNPs could possibly attribute to lowered therapeutic dosage and improved efficacy. This release profile corroborated with the results of fungicidal and polymicrobial biofilm inhibition assays. The synthesized MSNPs were stable up to 4 months at physiological pH as evident from the DLS analysis (data not shown).

### Antifungal activity

Candiduria is prevalent in ~10% of intensive care catheterized patients, where *C*. *albicans* was present in 69.1% of candiduria cases, forming a prevalent cause for catheter-associated candiduria^10^. In the current study, PCN and PCN-MSNPs were evaluated for their antifungal activity and their MICs were determined and depicted in Table 1. Miconazole (MZ) was used as standard control. The purified PCN exhibited good antifungal activity against test *Candida* strains at concentrations ranging between 7.8 µg mL^−1^ (34.94 µM) to 15.6 µg mL^−1^ (69.88 µM). PCN-MSNPs exhibited an improved antifungal activity at lower concentrations ranging between 1.9 to 3.9 µg mL^−1^ (8.51 to 17.47 µM), showed enhanced and persistent antifungal activity for extended time period of 124 h as compared to pure PCN which was potent up to 72 h only. This improved activity may be attributed to increased bioavailability of PCN due to increased surface area distribution and sustained release of PCN from PCN functionalized MSNPs. Moreover, PCN-MSNPs exhibited promising antifungal activity similar to that of standard MZ.

**Table 1:** Antifungal and fungicidal activity of native MSNPs, PCN and PCN-MSNPs.

Yeast strains	PCN (µM)		PCN-MSNPs (µM)		MSNPs (µM)	Miconazole (µM)	
	MIC	MFC	MIC	MFC	MIC	MIC	MFC
*Candida albicans* MTCC 183	69.8	139.76	17.4	34.9	—	9.3	18.7
*C*. *albicans* MTCC 227	34.9	69.8	8.5	17.4	—	9.3	18.7
*C*. *albicans* MTCC 1637	34.9	69.8	8.5	17.4	—	9.3	18.7
*C*. *albicans* MTCC 3017	34.9	69.8	8.5	17.4	—	9.3	18.7
*C*. *albicans* MTCC 3018	34.9	69.8	8.5	17.4	—	9.3	18.7
*C*. *albicans* MTCC 4748	34.9	69.8	17.4	34.9	—	9.3	18.7
*C*. *albicans* MTCC 7315	34.9	69.8	8.5	17.4	—	9.3	18.7
*C*. *parapsilosis* MTCC 1744	34.9	69.8	17.4	34.9	—	9.3	18.7
*C*. *glabrata* MTCC 3019	69.8	139.76	17.4	34.9	—	9.3	18.7
*C*. *krusei* MTCC 3020	34.9	69.8	8.5	17.4	—	9.3	18.7
*Issatchenkia hanoiensis* MTCC 4755	34.9	69.8	8.5	17.4	—	9.3	18.7

MIC: Minimum inhibitory concentration is the least concentration of test compound that could prevent visible growth of a microorganism, either bacteria or fungi, followed by incubation; MFC: Minimum fungicidal concentration is the least concentration of test compound that results in no growth of fungi at all or a growth with less than three colonies or 99% killing efficacy; PCN: Phenazine-1-carboxamide; MSNPs: Mesoporous silica nanoparticles; PCN-MSNPs: PCN-functionalized MSNPs; “—“: No activity.

### Minimum fungicidal concentration (MFC)

PCN and PCN-MSNPs (250 to 0 μg mL^−1^; 1.11 to 0 mM) were evaluated for their minimum fungicidal concentration against various *Candida* strains in Muller-Hinton broth (MHB). Purified PCN showed fungicidal activity within the range of 15.6 to 31.2 µg mL^−1^ (69.88 to 139.76 µM) against test *Candida* strains. The MFC exhibited by PCN-MSNPs against these same strains was between 7.8 to 15.6 µg mL^−1^ (34.74 to 69.88 µM), which was two-fold lower as compared to that of PCN. In addition, the activity of PCN-MSNPs was similar to the standard MZ. Based on the results, it was evident that PCN-MSNPs were promising as compared to PCN alone and equipotent as that of MZ with regard to antifungal activity. The results to this regard are depicted in Table 1.

### Polymicrobial growth inhibition

An antibiogram showing the effect of PCN and PCN-MSNPs on mixed populations of *C*. *albicans* MTCC 227 + *S*. *aureus* MTCC 96 and *C*. *albicans* MTCC 227 + *S*. *aureus* MLS-16 MTCC 2940 was plotted with respect to diameter of zone of inhibition as a function of time. The results showed that both PCN and PCN-MSNPs inhibited the growth of mixed pathogens, and the inhibitory effect of the later was more prominent and stable up to 120 h as apparent from the zone of inhibition (Supplementary Table S1). This improved activity of PCN-MSNPs could be due to the stable and controlled release of PCN under acidic growth conditions of *C*. *albicans* which was similar to the *in vitro* release profile at pH 5.2. These results suggest that PCN-MSNPs could effectively inhibit mixed populations of *C*. *albicans* MTCC 227 and the two *S*. *aureus* strains under *in vitro* conditions. The antibiogram results are depicted in Supplementary Fig. S6.

### Inhibition of *Candida* biofilms

Biofilms formed by various *Candida* species contribute to almost 15% of blood stream- and medical device-associated infections^44–46^. In the present study, the effect of PCN on biofilms formed by various *Candida* strains was studied and the results showed that PCN effectively inhibited these biofilms at a lower dose. Both the purified PCN and PCN-MSNPs were evaluated for their biofilm inhibition activity against *C*. *albicans* MTCC 227, *C*. *albicans* MTCC 1637, *C*. *albicans* MTCC 3017, *C*. *albicans* MTCC 3018, *C*. *albicans* MTCC 4748, *C*. *albicans* MTCC 7315, *C*. *parapsilosis* MTCC 1744, *C*. *glabrata* MTCC 3019, *C*. *krusei* MTCC 3020 and *I*. *hanoiensis* MTCC 4755, with MZ drug run in parallel as a standard control. The results of *Candida* biofilm inhibition assay are shown in Table 2. The purified PCN exhibited good inhibition of various *Candida* biofilms with inhibitory concentration ranging from 80–95 µM. Whereas, PCN-MSNPs exhibited promising inhibition of the test *Candida* biofilms at a lower dose ranging between 34 to 44 µM as compared to that of purified PCN. The above results suggest that PCN-MSNPs effectively inhibited biofilms formed *in vitro* by the test *Candida* strains.

**Table 2:** *Candida* biofilm inhibition by native MSNPs, PCN and PCN-MSNPs.

Yeast strains	Biofilm inhibitory concentration (BIC, µM)			
	PCN	PCN-MSNPs	MSNPs	Miconazole
*C*. *albicans* MTCC 183	83.20 ± 0.06	43.06 ± 0.32	—	27.22 ± 0.05
*C*. *albicans* MTCC 227	90.24 ± 0.11	38.18 ± 0.08	—	20.15 ± 0.11
*C*. *albicans* MTCC 1637	75.22 ± 0.19	35.12 ± 0.22	—	24.16 ± 0.25
*C*. *albicans* MTCC 3017	34.02 ± 0.10	31.00 ± 0.02	—	18.00 ± 0.09
*C*. *albicans* MTCC 3018	95.52 ± 0.09	37.27 ± 0.20	—	20.48 ± 0.18
*C*. *albicans* MTCC 4748	90.15 ± 0.10	38.18 ± 0.24	—	24.78 ± 0.13
*C*. *albicans* MTCC 7315	83.45 ± 0.20	44.11 ± 0.24	—	28.48 ± 0.23
*C*. *parapsilosis* MTCC 1744	80.15 ± 0.16	39.26 ± 0.22	—	22.19 ± 0.15
*C*. *glabrata* MTCC 3019	82.22 ± 0.16	40.13 ± 0.12	—	24.09 ± 0.20
*C*. *krusei* MTCC 3020	89.47 ± 0.11	34.28 ± 0.06	—	23.11 ± 0.17
*Issatchenkia hanoiensis* MTCC 4755	88.21 ± 0.11	36.14 ± 0.09	—	21.41 ± 0.17

BIC: Biofilm inhibitory concentration is the least concentration of test compound at which OD_600_ difference or less than 10% of the mean of two positive growth readings; PCN: Phenazine-1-carboxamide; MSNPs: Mesoporous silica nanoparticles; PCN-MSNPs: PCN-functionalized MSNPs; “–”: No activity.

### Polymicrobial biofilm inhibition

Under clinical situations, mixed populations of *C*. *albicans* and various bacteria coexist as biofilms in patients, which make the treatment very difficult and contributes to an increase in financial burden on hospitals and patients^6^. In the current study, *C*. *albicans* MTCC 227 + *S*. *aureus* MTCC 96 and *C*. *albicans* MTCC 227 + *S*. *aureus* MLS-16 MTCC 2940 biofilms were tested for their susceptibility to PCN and PCN-MSNPs (0–200 µM). The biofilm inhibitory effect of PCN-MSNPs was promising at a concentration of 53 µM as compared to PCN which was effective at 105 µM against biofilms of *C*. *albicans* MTCC 227 + *S*. *aureus* MTCC 96. On the other hand, *C*. *albicans* MTCC 227 + *S*. *aureus* MLS-16 MTCC 2940 biofilms were inhibited at a concentration of 55 µM by PCN-MSNPs, while pure PCN alone was effective at 119 µM (Table 3). This enhanced performance of PCN-MSNPs could be due to improved bioavailability and controlled release of PCN at acidic conditions of *C*. *albicans* growth as indicated by *in vitro* release studies.

**Table 3:** *Candida-Staphylococcus* polymicrobial biofilm inhibition by native MSNPs, PCN and PCN-MSNPs.

Yeast strains	Biofilm inhibitory concentration (µM)		
	PCN	PCN-MSNPs	MSNPs
*C*. *albicans* MTCC 227 + *S*. *aureus* MTCC 96	105.23 ± 0.14	53.23 ± 0.10	—
*C*. *albicans* MTCC 227 + *S*. *aureus* MLS-16 MTCC 2940	119.98 ± 0.22	55.64 ± 0.24	—

BIC: Biofilm inhibitory concentration is the least concentration of test compound at which OD_600_ difference or less than 10% of the mean of two positive growth readings. PCN: Phenazine-1-carboxamide; MSNPs: Mesoporous silica nanoparticles; PCN-MSNPs: PCN-functionalized MSNPs; “–”: No activity.

### Reduction in ergosterol content

**Figure 4:**
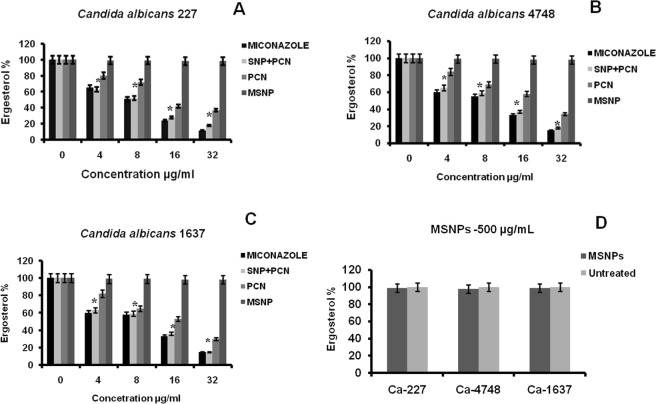
Ergosterol quantification in native MSNP, PCN and PCN-MSNPs treated *Candida* strains showing reduction in ergosterol content by PCN-MSNP (8 µg mL^−1^; 35.83 µM) as compared to standard, Miconazole. (**A**) *C*. *albicans* MTCC 227; (**B**) *C*. *albicans* MTCC 4748; (**C**) *C*. *albicans* MTCC 1637. (**D**) Reduction of ergosterol content by MSNPs at 500 µg mL^−1^ (2.23 mM). There was no growth observed in any of the *C*. *albicans* strains tested at 500 µg mL^−1^ (2.23 mM) of PCN or PCN-MSNPs. Untreated strains are used as control group (0 µg mL^−1^). In Fig. 4(A–C), * indicates statistical significance (*p* = 0.0001, α = 0.05), by one way ANOVA.

Ergosterol is mainly essential to maintain fungal cell membrane integrity and fluidity. Many of antifungals like amphotericin B interacts with ergosterol, altering the selective permeability of the cell membrane^47^. In the current study, it was noticed that ergosterol content was 100% in the untreated *Candida* strains, namely *C*. *albicans* MTCC 227, *C*. *albicans* MTCC 4748, *C*. *albicans* MTCC 1637, while a significant dose-dependent decrease in the ergosterol content was observed in case of both PCN and MZ-treated strains. As the dose of the PCN increased from 0 to 20 µg mL^−1^ (89.59 µM), resulted in decreased ergosterol content, which is a key component of the fungal cell membrane as indicated by the decline in peak heights. The UV-visible scans showed a flat line at the effective dose of PCN against *C*. *albicans* MTCC 227, *C*. *albicans* MTCC 4748 and *C*. *albicans* MTCC 1637 strains suggesting no detectable levels of sterols. The results to this regard are shown in Fig. 4. Our findings suggest that PCN alters the sterol profile and could be possibly exerting this antifungal activity by reducing the total ergosterol content, cell membrane disruption and subsequent cell death.

### Intracellular ROS generation in *C*. *albicans* MTCC 227

**Figure 5:**
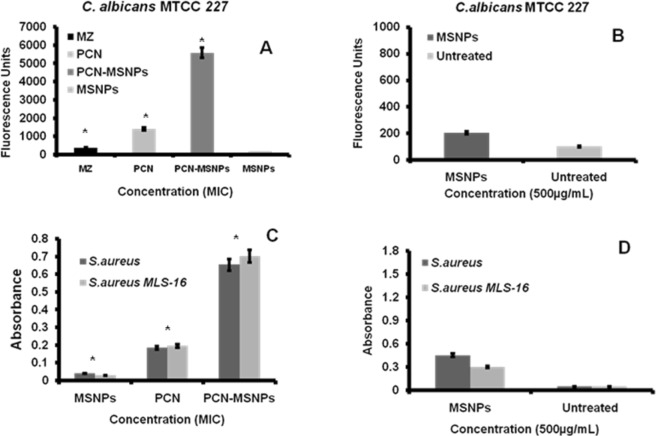
(**A**) PCN induced intracellular ROS accumulation in 24 h biofilm of *C*. *albicans* MTCC 227 measured by DCF-DA fluorometric assay. Percentage of intracellular ROS accumulation is directly proportional to fluorescence units. Amount of intracellular ROS accumulation by PCN was slightly higher than that of Miconazole at 3.9 µg mL^−1^ (17.47 µM). (**B**) Amount of intracellular ROS accumulation induced by native MSNPs (500 µg mL^−1^) in *C*. *albicans* MTCC 227, where MSNPs did not induce detectable intracellular ROS accumulation; (**C**) PCN at 3.9 µg mL^−1^ (17.47 µM) induced intracellular ROS accumulation in 24 h biofilms of *S*. *aureus* MTCC 96 and *S*. *aureus* MLS-16 MTCC 2940 measured by NBT assay. Percentage of intracellular ROS accumulation is directly proportional to absorbance recorded. (**D**) Amount of intracellular ROS accumulation induced by native MSNPs (500 µg mL^−1^) in both *Staphylococcus* biofilms. In the Fig. 5A,C, * indicates a statistical significance (*p* = 0.0001, α = 0.05) in ROS accumulation of treatment groups, by one way ANOVA.

Reactive oxygen species (ROS) is an important inducer of apoptosis in yeast cells^48,49^. Moreover, the oxidative stress in different *Candida* species results in lipid peroxidation, cell wall damage, reduced antioxidant capacity, protein carbonylation and catalase activities that are biologically significant^50^. ROS induced oxidative damage in *Candida* cells is one of the crucial mechanism of many antifungal agents. In order to understand any role played by PCN in oxidative damage of *C*. *albicans* MTCC 227, the intracellular ROS accumulation within the MSNPs treated *C*. *albicans* MTCC 227 sessile cells of mature biofilms and biofilms treated with PCN, MSNPs, PCN-MSNPs and MZ (MIC) was measured using fluorimetric 2′,7′-dichlorofluorescein diacetate (DCF-DA) assay. In this assay, the dye conversion is dependent on the metabolic and redox state of cells in the biofilm. The PCN-MSNPs treated biofilms exhibited approximately three folds higher to that of ROS accumulation as compared to that exhibited by the MZ treated counterparts. While the ROS accumulation exhibited by PCN-MSNPs treated were nearly two folds higher to that of ROS accumulation as compared to that exhibited by the PCN treated counterparts. The MSNPs treated biofilms showed negligible fluorescence, whereas PCN and MZ treated counterparts exhibited high fluorescence intensities which corresponded to increased levels of intracellular ROS accumulation. The fluorescence intensities of PCN and MZ-treated biofilms were almost similar indicating the efficient intracellular ROS accumulation caused by PCN (Fig. 5A). On the other hand, native MSNPs (500 µg/mL) treated and untreated *C*. *albicans* MTCC 227 biofilms showed a negligible amount of ROS accumulation (Fig. 5B).

### ROS assay in *S*. *aureus* MTCC 96 and *S*. *aureus* MLS-16 MTCC 2940

Most of the antibacterial agents exhibit intracellular ROS accumulation^51^. Reactive oxygen species accumulated in bacterial cells cause damage to DNA, RNA, proteins and lipids resulting in cell death because of their highly reactive nature and the presence of unpaired electrons. The inhibitory mechanism of PCN, MSNPs and PCN-MSNPs in *S*. *aureus* MTCC 96 and *S*. *aureus* MLS-16 MTCC 2940 was delineated by nitroblue tetrazolium (NBT) based ROS assay, which enables spectrophotometric quantification of generated ROS, specifically the superoxide ions. It was observed that the absorbance at 570 nm was five-fold higher in PCN treated *S*. *aureus* MTCC 96 and *S*. *aureus* MLS-16 MTCC 2940 biofilms as compared to native MSNPs treated ones indicating the accumulation of superoxide ions generated due to action of PCN (Fig. 5C). The absorbance of PCN-MSNPs treated *S*. *aureus* MTCC 96 and *S*. *aureus* MLS-16 MTCC 2940 biofilms was almost two-folds higher as compared to the PCN treated counterparts. The absorbance was directly proportional to the amount of intracellular ROS accumulation by PCN. The amount of ROS accumulated by PCN was seven-fold higher than the control (MZ). On the other hand, native MSNPs (500 µg mL^−1^) treated and untreated *S*. *aureus* MTCC 96 and *S*. *aureus* MLS-16 MTCC 2940 biofilms showed a negligible amount of ROS accumulation (Fig. 5D). Also this accumulated ROS could possibly damage the cell membrane which could be the result of high redox potential of PCN^52^. Moreover, the amount of intracellular ROS accumulated with PCN-MSNPs treatment was almost fifteen fold more than that observed with MZ, and this value was higher as compared to the previously reported antifungal mechanisms of nanoparticle conjugated antifungal compounds, such as that of MZ-conjugated AgNPs^49^ and N-acetyl cysteine conjugated AgNPs^53^. PCN inhibited *S*. *aureus* MTCC 96 and *S*. *aureus* MLS-16 MTCC 2940 (MIC value 3.9 µg mL^−1^; 17.47 µM) as determined in our previous study^31^. Hence, PCN, MSNP and PCN-MSNP (3.9 µg mL^−1^; 17.47 µM) were used to treat both the *Staphylococcus* strains for 6 h. MSNP treatment served as control.

### Confocal scanning laser microscopy

**Figure 6:**
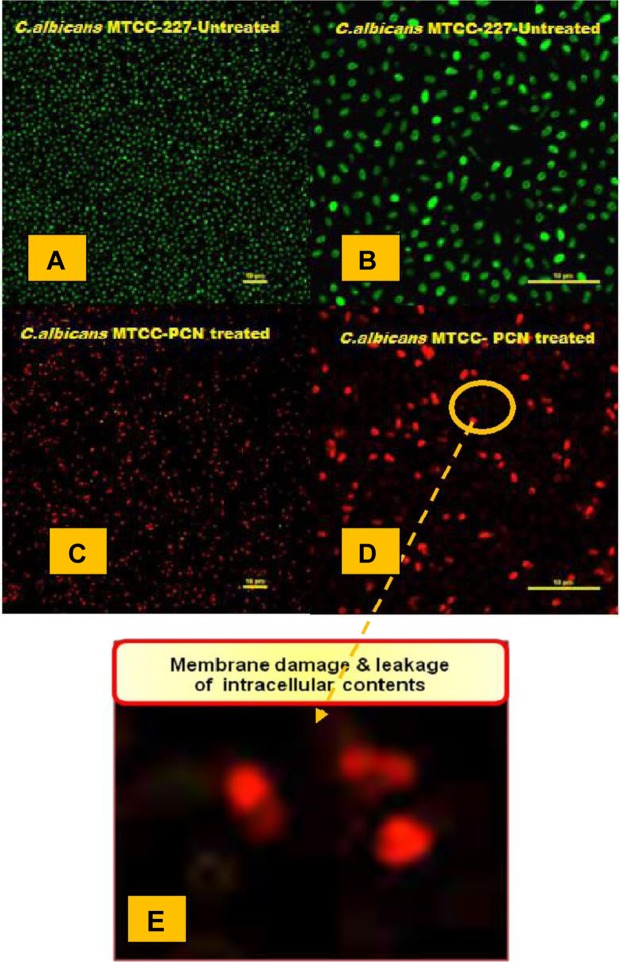
Confocal micrographs depicting membrane integrity status in 24 h grown cultures of *C*. *albicans* MTCC 227. (**A**,**B**) Untreated *C*. *albicans* MTCC 227 live cells with intact cell membrane as visualized by green staining with SYTO-9; (**C**,**D**) PCN-MSNPs (15.6 µg mL^−1^; 69.88 µM) treated *C*. *albicans* MTCC 227 dead cells with damaged cell membrane as visualized by red staining with propidium iodide; (**E**) PCN-MSNPs (15.6 µg mL^−1^; 69.88 µM) treated cells showing cell membrane disruption and leakage of intracellular contents.

Cell membrane in yeast cells plays an important role in structural and functional processes, the damage of which leads to lysis and cell death^54^. In the present study, PCN reduced the total content of ergosterol, that is crucial for the cell membrane integrity of *C*. *albicans* MTCC 227 which was assessed by LIVE/DEAD^®^ *Bac*Light^™^ kit and visualized by CSLM. The LIVE/DEAD BacLight kit was used to monitor the viability of *C*. *albicans* MTCC 227 cells as a function of cell membrane integrity of the cells which uses appropriate mixtures of SYTO 9, a green fluorescent nucleic acid dye and propidium iodide, a red fluorescent nucleic acid dye. SYTO 9 is permeable to the live *C*. *albicans* MTCC 227 cells having intact cell membranes and thus stains them green, while the propidium iodide stains red the *C*. *albicans* MTCC 227 cells having damaged cell membranes that were considered to be dead or in dying state. Under CSLM, the micrographs were thus observed as green and red fluorescent images for respective live and dead *C*. *albicans* MTCC 227 cells (Fig. 6). PCN-MSNPs significantly reduced the percentage of live *C*. *albicans* MTCC 227 cells at a concentration of 15.6 µg mL^−1^ (69.88 µM) as compared to the untreated cells. CSLM images suggest that PCN-MSNPs damaged the structural integrity of *C*. *albicans* MTCC 227 cells and caused yeast cell death which correlates well with the decrease in ergosterol content.

### Elemental analysis

ICP-OES was employed to analyze the levels of metal ions released during ion leakage (Supplementary Table S2). From the results, it was noticed that the concentration of metal ions such as Na^+^, K^+^ and Ca^2+^ released were in higher amounts (463.5, 293.1 and 380.4 ppm, respectively) in the PCN-treated *C*. *albicans* MTCC 227 cells as compared to the untreated control (300.2, 47.47 and 18.40 ppm, respectively), which indicates that PCN caused membrane damage resulting in Na^+^, K^+^ and Ca^2+^ leakage and subsequent cell death.

### Anti-biofilm activity of PCN-MSNPs coating on silicone urethral catheter

Among the medical devices, urinary catheters form a very suitable surface for rapid microbial colonization, adhesion and biofilm growth making their removal as a sole choice for preventing the further spread of infection^55^. *S*. *aureus* is one of the frequent causative agent for the urinary catheter associated infections in patients^56^. Among fungi, *C*. *albicans* is a major causative agent for urinary catheter-associated infections in intensive care patients^10^. With the emergence of resistant *C*. *albicans* and *S*. *aureus* strains, the treatment and management of *Candida-Staphylococcus* mixed biofilms has become more challenging^56^. Considering the facts, the novel compounds that can specifically target and inhibit the biofilm formation would be interesting as compared to the rational use of antibiotics. The PCN-MSNPs anti-biofilm coating was effective in inhibiting preformed mixed biofilms of *C*. *albicans* MTCC 227 and *S*. *aureus* MTCC 96. It was reported earlier that surface charge and hydrophobicity properties plays a key functional role which influenced the surface chemistry of the coating material and prevents the bacterial attachment and biofilm formation^57^. Various clinical manifestations like oral and ear infections, diabetic wound infections, pulmonary and urinary tract infections as well as medical implant associated infections are largely caused by biofilms formed by multi-species microbial communities that exhibit high resistance to antimicrobial agents which is significant from a clinical perspective^8,58^.

**Figure 7:**
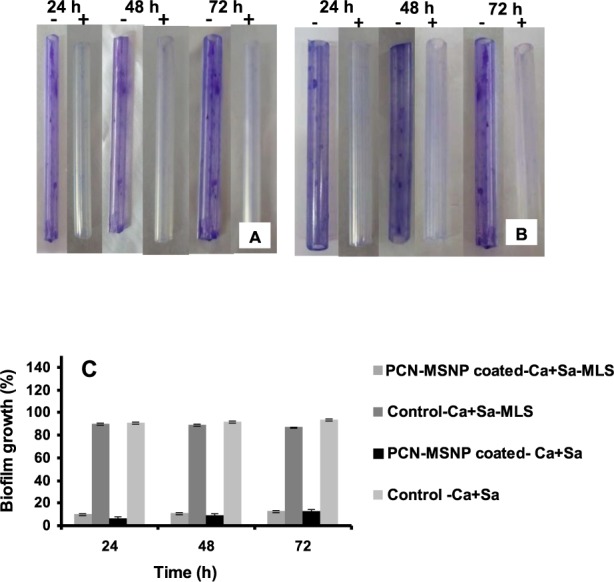
Performance of PCN-MSNPs coating on silicone urethral catheter as a function of incubation time. PCN-MSNPs coated (+) and uncoated (−) catheters were incubated in overnight grown mixed cultures of (**A**) *C*. *albicans* MTCC 227 + *S*. *aureus* MTCC 96 and (**B**) *C*. *albicans* MTCC 227 + *S*. *aureus* MLS-16 MTCC 2940. The biofilm formation in A and B group catheters after 24, 48 and 72 h incubation were visualized and quantified using crystal violet staining assay. (**C**) Polymicrobial biofilm inhibition by PCN-MSNPs coatings on silicone urethral catheters as a function of time. In (**C**), * indicates the statistically significant difference (*p* = 0.0001, α = 0.05) in biofilm growth percentage among the treatment at each time point, by one way ANOVA.

In the present study, PCN-MSNPs were coated on silicone urethral catheters and were further evaluated for their performance as anti-biofilm coating agents. The coating of PCN-MSNPs over silicone catheters could be the result of surface modification by functionalization of PCN moieties which prevents the attachment of mixed biofilms. The successful coating was confirmed by change of color on the catheter surface (Supplementary Fig. S7). The performance of anti-biofilm coatings of PCN-MSNPs revealed that mixed biofilms of *C*. *albicans* MTCC 227*-S*. *aureus* MTCC 96 and *C*. *albicans* MTCC 227*-S*. *aureus* MLS-16 MTCC 2940 were effectively inhibited as a function of time as illustrated in Fig. 7A,B. The anti-biofilm assay showed that mixed biofilm formation of *C*. *albicans* MTCC 227*-S*. *aureus* MTCC 96 on coated catheters was reduced by 89.1%, 88.2% and 88.1% at 24, 48 and 72 h of catheter incubation, respectively, while the mixed biofilm formation of *C*. *albicans* MTCC 227*-S*. *aureus* MLS-16 MTCC 2940 was reduced by 93.8%, 92% and 91% at 24, 48 and 72 h of catheter incubation, respectively (Fig. 7C).

### *In vitro* cytotoxicity

The *in vitro* cytotoxic effect of PCN-MSNPs was evaluated in NIH-3T3 cell line based on MTT assay, which indicated that PCN-MSNPs were non-toxic up to IC_50_ value of 100 µM (Supplementary Fig. S8). While the antifungal activity and polymicrobial biofilm inhibition by PCN-MSNPs were exhibited at a three-fold lower concentrations than the IC_50_ value in MTT assay. All the above results suggest that PCN-MSNP coatings could serve as promising antimicrobial agents to combat CAUTI. Further, *in vivo* studies could possibly transform the PCN-MSNP application under healthcare situations. This is the first report on PCN-MSNPs for use as antimicrobial coatings against microbial adhesion and biofilm formation on silicone urethral catheters.

In conclusion, we hypothesize that PCN induced ROS accumulation and reduction in ergosterol content could damage the cell membrane resulting in the disregulation of intracellular concentrations of key ions such as Na^+^, K^+^ and Ca^2+^, thus breaking the osmotic balance of the cell. Leakage of these ions may cause cell lysis and death in view of their crucial roles in activating various biologically significant enzymes involved in respiration and biosynthetic pathways^59^. Moreover, the membrane in *Candida albicans* is composed of various sterols and lipids that act as barriers for various components and ions apart from the forming matrix for various significant proteins^60,61^. The disturbance in any of these lipids or sterols results in efflux of ions^62^. This hypothesis was proved based on confocal micrographs that clearly reveal the cell membrane damage and lysis leading to leakage of intracellular cell contents. For understanding the status of ionic homeostasis, the PCN treated and untreated *C*. *albicans* MTCC 227 cell supernatants were subjected to elemental analysis. The results of elemental analysis indicated that PCN treated cells showed significant leakage of Na^+^, K^+^ and Ca^2+^ ions as compared to control groups.

## Supplementary information

Supplementary information
