# Supplementary material for: Phenazine-1-carboxamide functionalized mesoporous silica nanoparticles as antimicrobial coatings on silicone urethral catheters
Source: Sci Rep. 2019 Apr 17;9:6198. doi: 10.1038/s41598-019-42722-9 (PMC6470230; doi:10.1038/s41598-019-42722-9)

**Supplementary Information**

**Phenazine-1-carboxamide functionalized mesoporous silica nanoparticles as antimicrobial coatings on silicone urethral catheters**

K. Sirisha^a,c^, Sudhakar Jinka^b^, Nagaprasad Puvvada^b^, Rajkumar Banerjee^b,c^ and C. Ganesh Kumar^a,c^,*

^a^Department of Organic Synthesis and Process Chemistry, CSIR-Indian Institute of Chemical Technology, Uppal Road, Hyderabad 500007, India.

^b^Department of Applied Biology, CSIR-Indian Institute of Chemical Technology, Uppal Road, Hyderabad 500007, India.

^c^Academy of Scientific and Innovative Research, Ghaziabad 201002, India.

**Running Title**: Phenazine-1-carboxamide functionalized silica nanoparticles

^*^Corresponding author. Tel : +91-40-27193105, +91-40-27193189

Email address : [cgkumar.iict@gov.in](mailto:cgkumar.iict@gov.in); [cgkumar5@gmail.com](../cgkumar5@gmail.com)

**Table S1**. Antimicrobial activity of MSNPs

| Pathogen | Diameter of zone of inhibition (in mm) | | |
| --- | --- | --- | --- |
|  | MSNPs | PCN | PCN-MSNPs |
| *Candida albicans* MTCC 183 | 2 | 12 | 23 |
| *C. albicans* MTCC 227 | 2 | 12 | 23 |
| *C. albicans* MTCC 1637 | 2 | 13 | 23 |
| *C. albicans* MTCC 3017 | 2 | 12 | 24 |
| *C. albicans* MTCC 3018 | 1.6 | 12 | 23 |
| *C. albicans* MTCC 4748 | 2 | 13 | 22 |
| *C. albicans* MTCC 7315 | 2.3 | 13 | 23 |
| *C. glabrata* MTCC 3019 | 2 | 13 | 23 |
| *C. krusei* MTCC 3020 | 2 | 13 | 21 |
| *Issatchenkia hanoiensis* MTCC 4755 | 2 | 13 | 23 |

**Table S2.** ICP-OES analysis of PCN treated *Candida albicans* MTCC 227

| Concentration (ppm) | *C. albicans* MTCC 227  (Untreated) | *C. albicans* MTCC 227  (PCN treated, 15.6 µg mL^-1^) |
| --- | --- | --- |
| Na ^+^ | 300.2 | 463.5 |
| K ^+^ | 47.47 | 293.1 |
| Ca^2+^ | 18.40 | 380.4 |

**Supplementary Figure Legends**

**Figure S1**. Low angle powder XRD pattern of synthesized mesoporous silica nanoparticles. The characteristic peak of mesoporous silica nanoparticles with a 2θ value of 2.1^o^ corresponding to (100) facet peak of hexagonally ordered pore channeling.

**Figure S2**. Dynamic light scattering analysis of MSNPs and PCN-MSNPs (A) Pore size distribution curve of synthesized MSNPs showing average particle diameter as 193 nm (B) Pore size distribution curve of synthesized PCN functionalized MSNPs showing average particle diameter as 234 nm

**Figure S3**. Thermogravimetric analysis (TGA) graph of MSNPs (S1) and PCN-MSNPs (S2)

**Figure S4**. UV absorption spectra of (a) Purified phenazine-1-carboxamide showing two characteristic peaks at 289 and 431 nm. (b) Synthesized silica nanoparticles (c) Phenazine-1-carboxamide functionalized to mesoporous silica nanoparticles showing a shift in the two characteristic peaks.

**Figure S5**. *In vitro* release profile of phenazine-1-carboxamide (PCN) from PCN functionalized mesoporous silica nanoparticles (PCN-MSNPs) at two pH conditions, pH 5.2 and pH 7.4. At both these pH conditions, the maximum release of PCN was 80% which was achieved in 36 h.

**Figure S6**. Antibiogram showing effect of phenazine-1-carboxamide (PCN) and phenazine-1-carboxamide functionalized mesoporous nanoparticles (PCN-MSNPs) on the growth of mixed populations of *Candida albicans* MTCC 227 + *Staphylococcus aureus* MTCC 96; *Candida albicans* MTCC 227 + *Staphylococcus aureus* MLS-16 MTCC 2940. The PCN-MSNPs showed sustained inhibition due to the action of the combination of *Candida albicans* MTCC 227 + *Staphylococcus aureus* MTCC 96 up to 120 h as compared to short term inhibition by PCN alone as evident from zone of inhibition. The PCN-MSNPs also showed sustained inhibition due to the action of the combination of *Candida albicans* MTCC 227 + *Staphylococcus aureus* MLS-16 MTCC 2940 up to 120 h as compared to short term inhibition by PCN as evident from zone of inhibition in agar well diffusion assay.

**Figure S7**. Silicone urethral catheters (a) Uncoated and (b) PCN-MSNPs coated

**Figure S8.** Cytotoxicity of PCN-MSNPs against NIH-3T3 fibroblasts

Figure S1


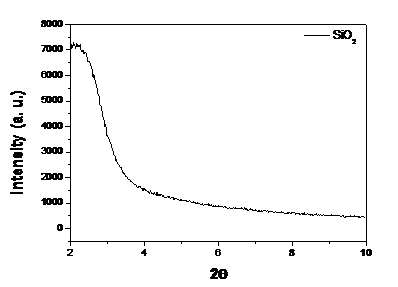


Figure S2


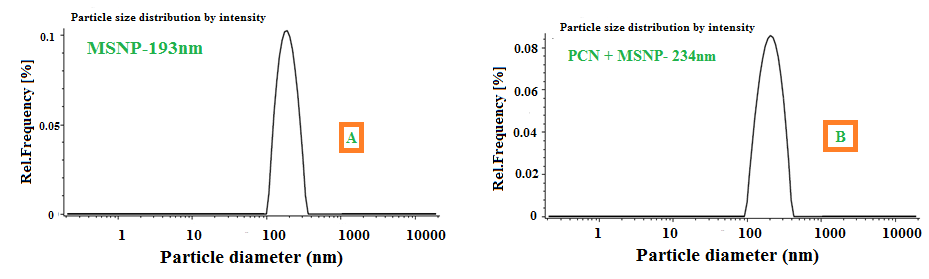


Figure S3


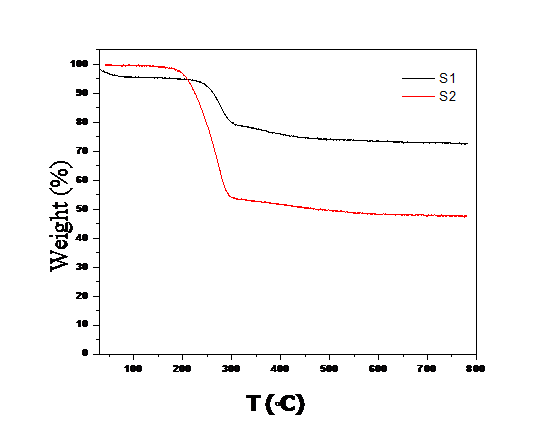


Figure S4


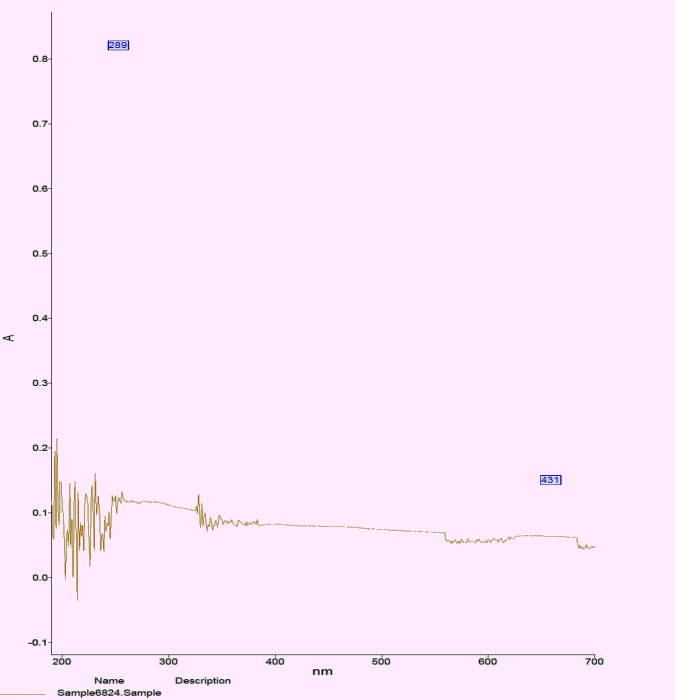


**B**

**305 nm**


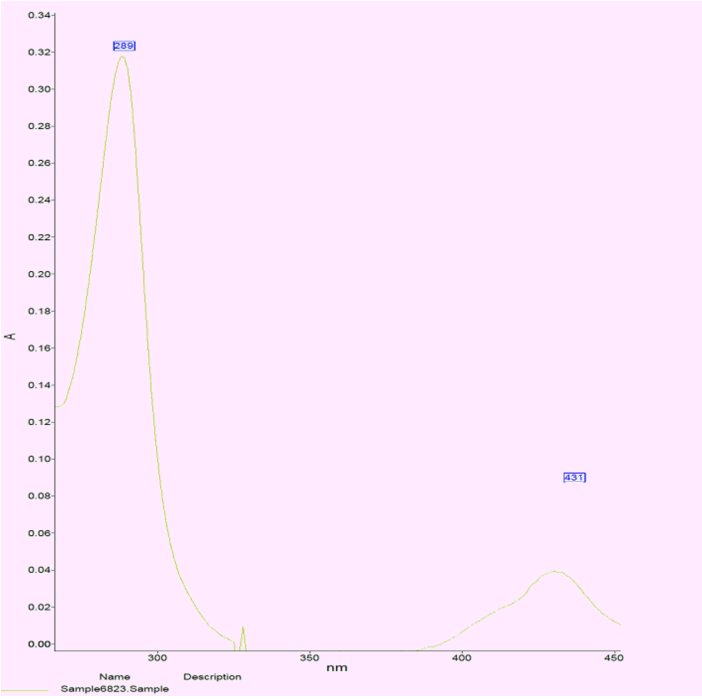


**A**

**294 nm**


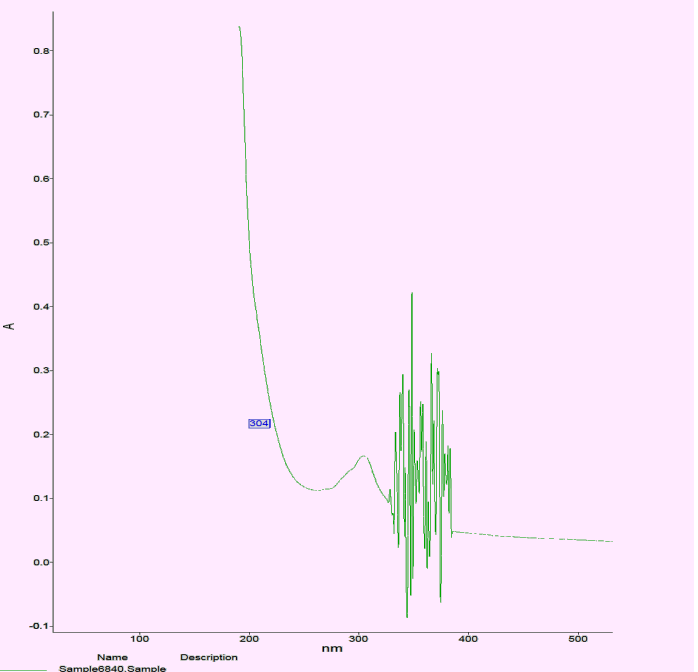


**431 nm**

**C**

Figure S5

Figure S6


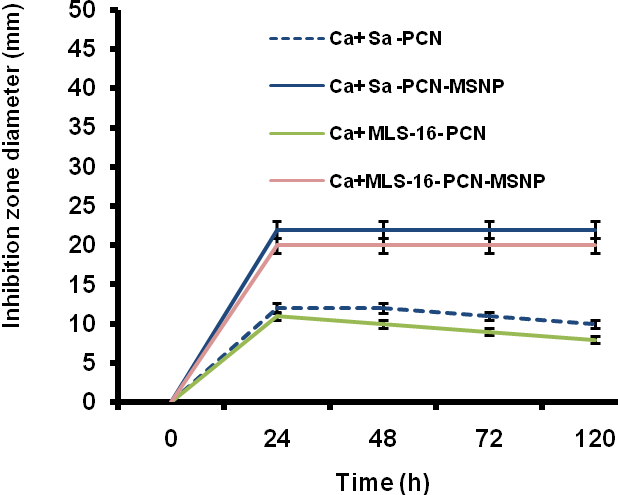


Figure S7


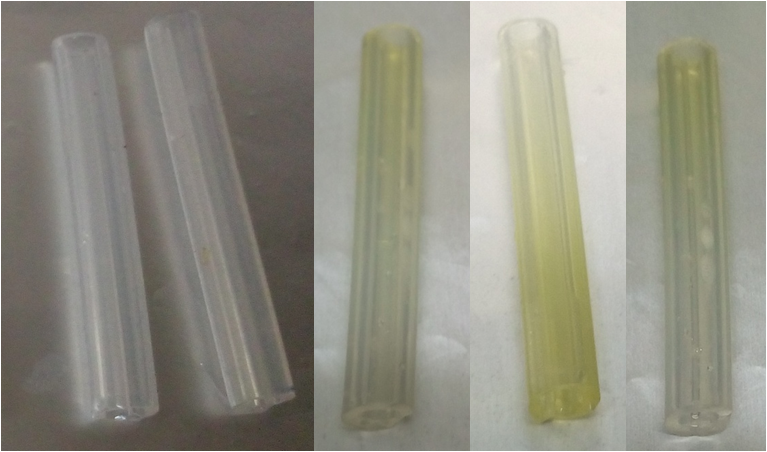


**A**

**B**

Figure S8


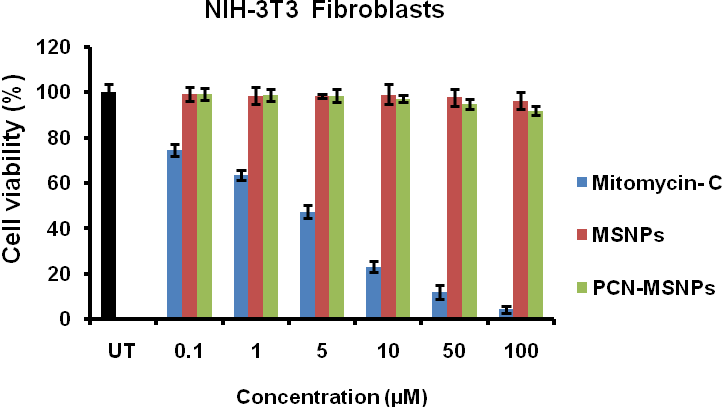

Supplement: Supplementary file 1 — Supplementary information [file 41598_2019_42722_MOESM1_ESM.doc]
